# Clinical features and prognosis of ANCA-associated vasculitis patients who were double-seropositive for myeloperoxidase-ANCA and proteinase 3-ANCA

**DOI:** 10.1007/s10238-024-01318-y

**Published:** 2024-04-02

**Authors:** Yizi Gong, Chanjuan Shen, Ting Meng, Wei Lin, Xueling Hu, Rong Tang, Qi Xiong, Joshua D. Ooi, Peter J. Eggenhuizen, Jinbiao Chen, Ya-Ou Zhou, Hui Luo, Jia Xu, Ning Liu, Ping Xiao, Xiangcheng Xiao, Yong Zhong

**Affiliations:** 1 Department of Nephrology, Xiangya Hospital, Central South University, 87 Xiangya Road, Changsha, Hunan Province China; 2 Key Laboratory of Biological, Nanotechnology of National Health Commission, Xiangya Hospital, Central South University, Changsha, Hunan China; 3 National Clinical Research Center for Geriatric Disorders, Xiangya Hospital, Central South University, Changsha, 410008 Hunan China; 4 Department of Hematology, The Affiliated Zhuzhou Hospital of Xiangya Medical College, Central South University, Zhuzhou, China; 5 Department of Pathology, Xiangya Hospital, Central South University, Changsha, Hunan Province China; 6 Centre for Inflammatory Diseases, Department of Medicine, School of Clinical Sciences, Monash University, Clayton, VIC Australia; 7 Department of Medical Records and Information, Xiangya Hospital, Central South University, Changsha, Hunan Province China; 8 Department of Rheumatology and Immunology, Xiangya Hospital, Central South University, Changsha, China

**Keywords:** ANCA-associated vasculitis, Double-positive vasculitis, Myeloperoxidase, Proteinase 3, Prognosis

## Abstract

Anti-neutrophil cytoplasmic antibody (ANCA)–associated vasculitis (AAV) patients with dual positivity for proteinase 3-ANCA (PR3-ANCA) and myeloperoxidase-ANCA (MPO-ANCA) are uncommon. We aimed to investigate these idiopathic double-positive AAV patients’ clinical features, histological characteristics, and prognosis. We reviewed all the electronic medical records of patients diagnosed with AAV to obtain clinical data and renal histological information from January 2010 to December 2020 in a large center in China. Patients were assigned to the MPO-AAV group or PR3-AAV group or idiopathic double-positive AAV group by ANCA specificity. We explored features of idiopathic double-positive AAV. Of the 340 patients who fulfilled the study inclusion criteria, 159 (46.76%) were female, with a mean age of 58.41 years at the time of AAV diagnosis. Similar to MPO-AAV, idiopathic double-positive AAV patients were older and had more severe anemia, lower Birmingham Vasculitis Activity Score (BVAS) and C-reactive protein (CRP) levels, less ear, nose, and throat (ENT) involvement, higher initial serum creatinine and a lower estimated glomerular filtration rate (eGFR) when compared with PR3-AAV (*P* < 0.05). The proportion of normal glomeruli of idiopathic double-positive AAV was the lowest among the three groups (*P* < 0.05). The idiopathic double-positive AAV patients had the worst remission rate (58.8%) among the three groups (*P* < 0.05). The relapse rate of double-positive AAV (40.0%) was comparable with PR3-AAV (44.8%) (*P* > 0.05). Although there was a trend toward a higher relapse rate of idiopathic double-positive AAV (40.0%) compared with MPO-AAV (23.5%), this did not reach statistical significance (*P* > 0.05). The proportion of patients who progressed to ESRD was 47.1% and 44.4% in the idiopathic double-positive AAV group and MPO-AAV group respectively, without statistical significance. Long-term patient survival also varied among the three groups (*P* < 0.05). Idiopathic double-positive AAV is a rare clinical entity with hybrid features of MPO-AAV and PR3-AAV. MPO-AAV is the “dominant” phenotype in idiopathic double-positive AAV.

## Introduction

Anti-neutrophil cytoplasmic antibody (ANCA)–associated vasculitis (AAV) is a group of autoimmune disorders characterized by inflammation and/or necrosis of small- to medium-sized blood vessels, endothelial injury, and tissue damage [1]. Microscopic polyangiitis (MPA), granulomatosis with polyangiitis (GPA), and eosinophilic GPA (EGPA) are three main types of AAV according to the 2012 Chapel Hill Consensus Conference [2]. As a hallmark of AAV, proteinase 3-ANCA (PR3-ANCA) or myeloperoxidase-ANCA (MPO-ANCA) can be detected. Although the classification of the clinical phenotype has been commonly used for AAV since the 1990 s, evidence supporting the classification based on the ANCA subtype has been growing [3–8].

Whether AAV patients who were double positive for PR3-ANCA and MPO-ANCA were distinguishable from single positivity AAV for PR3-ANCA or MPO-ANCA remains elusive. Hence, we aimed to explore the clinical features, histological characteristics, and prognosis of the rare idiopathic double-seropositive AAV patients in this study.

## Methods

### Study population

This retrospective analysis included patients diagnosed with AAV between January 2010 and December 2020 in Xiangya Hospital, China, according to the 2012 Chapel Hill Consensus Conference [2]. The classification criteria we used in this study are the 2022 American College of Rheumatology/European Alliance of Associations for Rheumatology classification criteria for AAV [9–11] and the Watt’s Algorithm for classifying AAV [12]. Standard indirect immunofluorescence assay (IFA) (Euroimmun, Lübeck, Germany) and direct enzyme-linked immunosorbent assay (ELISA) (Inova Diagnostics, San Diego, USA) were applied to detect the presence of ANCA. AAV patients who tested positive for both MPO-ANCA and PR3-ANCA were assigned to the double-positive AAV group. Before diagnosis, the tests were repeated at least once to avoid the diagnosis of double-positive on occasion. The tests were also repeated during the follow-up. The ANCA testing results of ELISA were paired with IFA. The cut-off levels for MPO-ANCA and PR3-ANCA titers were both 20 U/ml. Patients were assigned to the MPO-AAV group, PR3-AAV group, or idiopathic double-positive AAV group by ANCA specificity at the diagnosis, hereafter MPO-AAV, PR3-AAV, and idiopathic double-positive AAV. The exclusion criteria were as follows: (1) EGPA or secondary vasculitis, e.g., infective endocarditis; (2) the coexistence of other autoimmune disease or nephropathy; (3) patients positive for anti-glomerular basement membrane (GBM); (4) pregnancy, or previous malignancy; (5) activated hepatitis B virus, hepatitis C virus, or human immunodeficiency virus infection; (6) drug-induced AAV.

The last time we followed up with our patients was March 30, 2022. And the median time of follow-up was 27.33 months (range 0.13–134.10 months).

### Data retrieval

We reviewed all the eligible patients’ electronic medical records to obtain data and information, which contain baseline clinical characteristics, laboratory test data, systems involved, comorbidities, renal histological information, and therapeutic regimen. We used the Birmingham Vasculitis Activity Score (BVAS; version 3) to estimate disease activity [13]. Systems involvement was determined by biopsy or using previously described criteria [14, 15]. The original pathology records of kidney biopsy were collected and evaluated by two professional pathologists. The percentage of glomeruli with different features according to the presence of global sclerosis, crescents, and necrosis was calculated. Renal biopsies were classified into focal, crescentic, mixed, or sclerotic subgroups based on the Berden classification [16]. Glomerular features like fibrinoid necrosis, Bowman’s capsule rupture, granulomatous lesions, and thrombotic microangiopathy were also recorded. Arteriolar lesions were listed as fibrinoid necrosis. A semi-quantitative score was used to describe interstitial infiltrates and tubulointerstitial lesions, namely mild (score of 1) for < 25% renal tubulointerstitial involvement, moderate (score of 2) for 25–50% renal tubulointerstitial involvement, and severe (score of 3) for > 50% renal tubulointerstitial involvement [17]. To evaluate the intensity of C3, C4, C1q, IgA, IgG, and IgM in the renal biopsies, we scored them under an immunofluorescence microscope after staining with fluorescein-conjugated (FITC) antisera specific for C3, C4, C1q, IgA, IgG, IgM. They were listed as scale 0 for negative ( − ), scale 1 for trace ( ±) and mild (1 +), scale 2 for moderate (+ +), and scale 3 for strong (+ + +)[18]. The prognosis of renal risk was according to the renal risk score published by Brix et al. [19].

### Treatment

Management of AAV according to the KDIGO guidelines and EULAR/ERA-EDTA recommendations was administered to patients in induction remission and maintenance remission [20, 21]. For induction remission, patients received glucocorticoids together with cyclophosphamide or rituximab. Cyclophosphamide was administered intravenously 500–750 mg/m^2^ per month and the dosage was adjusted according to the count of leukocytes to maintain it above 4 × 10^9^/L. The rituximab dose was 375 mg/m^2^ of body surface area, once a week or 1000 mg twice a month for four infusions. Furthermore, plasma exchange and/or intravenous methylprednisolone pulses were also administered to some patients. During maintenance remission, the patients received azathioprine (1.5–2 mg/kg/d) or mycophenolate mofetil (250-1000 mg/d bid) in combination with low-dose glucocorticoids. The dosage of azathioprine or mycophenolate mofetil was adjusted by age, eGFR, side effects, and level of white blood cells.

### Definitions

ESRD was defined as a requirement for hemodialysis or peritoneal dialysis for over 3 months or kidney transplantation [22]. Remission was defined as the absence of typical signs, symptoms, or other features of active AAV with or without immunosuppressive therapy [23, 24]. Relapse was defined as the recurrence of active AAV after a period of remission [23, 24]. We define the time from treatment to the development of ESRD as renal survival and the time from treatment to death as patient survival. The estimated glomerular filtration rate (eGFR) was estimated by the CKD-EPI creatinine equation [25]. Disease duration was defined as the time from symptoms to diagnosis. Follow-up duration was defined as the time of initial treatment to the occurrence of death or the deadline of follow-up.

### Statistical analysis

We used SPSS Statistics v.26 (IBM Corp, Armonk, NY, USA) and GraphPad Prism v.9 (GraphPad Software, La Jolla, CA, USA) to analyze data. Shapiro–Wilk test was used to test normally distributed variables. Continuous data were presented as mean (± SD) or median (interquartile range, IQR). Categorical variables were expressed as frequency and percentage. Continuous data were compared by using ANOVA with post hoc LSD test or Kruskal–Wallis test. For the comparison of categorical variables, the χ2 test or Fisher’s exact test was applied. Kaplan–Meier curves depicted survival distribution and log-rank tests verified unadjusted survival differences. A two-sided *P* value < 0.05 was considered statistically significant.

## Results

### Demographics and subject characteristics

**Fig. 1:**
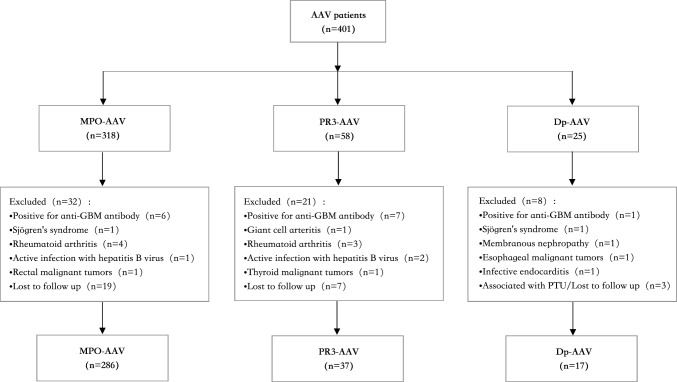
Flowchart of included patients

A total of 401 patients were included in this study between January 2010 and December 2020 in Xiangya Hospital (Fig. 1). 14 patients were excluded due to diagnosis of anti-GBM disease (1 out of 14 were dual-positive for MPO-ANCA and PR3-ANCA). 11 patients were excluded because of other autoimmune diseases or nephropathy (2 out of 11 were dual-positive for MPO-ANCA and PR3-ANCA, one was diagnosed with Sjögren's syndrome and the other one was diagnosed with membranous nephropathy). 7 patients were excluded due to previous malignancy and infections (2 out of 7 were dual-positive for MPO-ANCA and PR3-ANCA, one had esophageal malignant tumors before and the other one was combined with infective endocarditis). And 29 patients were lost during follow-up (3 out of 29 were dual-positive for MPO-ANCA and PR3-ANCA, these three patients were associated with propylthiouracil (PTU)). Finally, 340 patients were enrolled in our study.

Of the 340 patients who fulfilled the study inclusion criteria, 159 (46.76%) were female, with a mean age of 58.41 years at the time of AAV diagnosis. Among these 340 patients, 286 (84.1%) patients were in the MPO-AAV group, 37 (10.9%) patients in the PR3-AAV group, and 17 (5.0%) patients in the idiopathic double-positive group. Among the 17 idiopathic double-positive AAV patients, 16 patients were positive for p-ANCA while 1 patient was positive for c-ANCA. The average titer for MPO-ANCA of the 17 idiopathic double-positive AAV patients was 84.00 ± 39.26 U/ml. The median titer for PR3-ANCA of them was 38.87 U/ml, ranging from 30.15 to 190.49 U/ml. The clinical characteristics of the 3 groups are shown in Table 1. The age of idiopathic double-positive AAV patients (57.59 ± 14.97) was similar to MPO-AAV patients (60.55 ± 13.99) but were older than PR3-AAV patients (43.35 ± 16.66) (*P* < 0.05). No significant difference in sex distribution among the 3 groups was found in our study. Among 17 idiopathic double-positive patients, 2 (11.8%) were diagnosed with GPA, and 15 (88.2%) were diagnosed with MPA. We found no significant difference in the duration of disease when comparing idiopathic double-positive AAV with the other two groups.

**Table 1:** Baseline clinical characteristics and outcomes

	MPO-AAV (*n* = 286)	PR3-AAV (*n* = 37)	Double-positive AAV (*n* = 17)	*P* value
Age, year (mean, SD)	60.55 ± 13.99	43.35 ± 16.66^a^	57.59 ± 14.97^b^	< 0.0001
Female (%)	135 (47.2%)	13 (35.1%)	11 (64.7%)	0.121
*•Vasculitis type, n (%)*				
MPA	245 (85.7%)	5 (13.5%)	15 (88.2%)	< 0.0001
GPA	41 (14.3%)	32 (86.5%)	2 (11.8%)	
Duration of disease, d (median, IQR)	58 (31, 95)	89 (38, 327)^a^	74 (23, 150)	0.020
White blood cells, 10^9^/L (mean, SD)	9.37 ± 4.79	12.49 ± 7.09^a^	6.88 ± 3.00^a, b^	0.001
Neutrophil, 10^9^/L (mean, SD)	7.46 ± 4.50	9.22 ± 6.76	6.46 ± 4.97	0.227
Lymphocyte, 10^9^/L (mean, SD)	1.14 ± 0.63	1.17 ± 0.63	0.90 ± 0.46	0.129
Hemoglobin, g/L (mean, SD)	82.68 ± 20.04	95.49 ± 23.47^a^	74.15 ± 40.10^b^	0.010
Platelet, 10^9^/L (mean, SD)	246.25 ± 108.04	330.39 ± 170.52^a^	183.71 ± 80.06^a, b^	0.001
Serum albumin, g/L (mean, SD)	30.66 ± 6.04	38.05 ± 17.22^a^	35.46 ± 11.12^a^	0.016
Serum globulin, g/L (mean, SD)	31.06 ± 6.62	31.93 ± 6.96	33.13 ± 5.24	0.365
Proteinuria, g/24 h (median, IQR)	1.08 (0.65, 2.05)	0.83 (0.41, 1.91)	1.83 (0.94, 2.97)	0.435
Hematuria, *n* (%)	201 (70.3%)	27 (73.0%)	11 (64.7%)	0.826
Initial serum creatinine, μmol/L (median, IQR)	329.5 (161.775, 562)	104 (64, 215.2)^a^	473.4 (167, 659)^b^	< 0.0001
Initial eGFR, ml/min/1.73 m (median, IQR)	14 (7.3, 32.9025)	72.81 (35.495, 111.19)^a^	7.42 (6.82, 30.78)^b^	< 0.0001
ESR, mm/h (median, IQR)	67.5 (39.25, 106.75)	80 (49.25, 120)	47 (29.5, 93.50)	0.229
CRP, mg/L (median, IQR)	22.30 (5.11, 76.75)	74.80 (21.8, 128.50)^a^	8.94 (4.28, 67.75)^b^	< 0.0001
Serum C3, mg/L (mean, SD)	766.68 ± 220.80	902.93 ± 406.77	792.00 ± 156.79	0.215
Serum C4, mg/L (mean, SD)	238.25 ± 103.78	226.04 ± 131.80	234.94 ± 65.32	0.838
IgA, mg/L (mean, SD)	2441.47 ± 1182.56	2858.32 ± 1055.82	2255.31 ± 1052.27	0.151
IgG, g/L (mean, SD)	14.05 ± 4.61	14.65 ± 5.60	14.85 ± 5.44	0.677
IgM, mg/L (mean, SD)	1079.08 ± 664.70	1189.80 ± 721.96	968.25 ± 484.53	0.566
BVAS (mean, SD)	17.45 ± 5.94	19.73 ± 5.48^a^	14.71 ± 6.03^b^	0.011
No. of systems involved	3 (2, 3)	4 (3, 5)^a^	2 (2, 3)^b^	< 0.0001
Renal involvement, *n* (%)	271 (94.8%)	32 (86.5%)	16 (94.1%)	0.121
ENT involvement, *n* (%)	23 (8.0%)	12 (32.4%)^a^	1 (5.9%)^b^	< 0.0001
Lung involvement, *n* (%)	193 (67.5%)	27 (73.0%)	9 (52.9%)	0.343
*•Comorbidities*				
Hypertensive diseases, *n* (%)	111 (38.8%)	5 (13.5%)^a^	3 (17.6%)	0.003
Lung diseases, *n* (%)	105 (36.7%)	13 (35.1%)	4 (23.5%)	0.543
Type 2 diabetes mellitus, *n* (%)	24 (8.4%)	3 (8.1%)	1 (5.9%)	1.000
Coronary heart disease, *n* (%)	21 (7.3%)	3 (8.1%)	1 (5.9%)	0.905
Median follow-up time, months (median, IQR)	26.3 (12.11, 41.56)	39.37 (18.03, 65.67)^a^	33.33 (7.63, 63.18)	< 0.0001
Requirement for RRT at presentation, *n* (%)	79 (27.6%)	3 (8.1%)^a^	4 (23.5%)	0.036
*•Outcomes*				
Remission, *n* (%)	238 (83.2%)	29 (78.4%)	10 (58.8%)^a^	0.037
Relapse, *n* (%)	56 (23.5%)	13 (44.8%)^a^	4 (40.0%)	0.020
ESRD, *n* (%)	127 (44.4%)	8 (21.6%)^a^	8 (47.1%)	0.028
Death, *n* (%)	90 (31.5%)	5 (13.5%)^a^	2 (11.8%)	0.021

*MPO-AAV* Myeloperoxidase-anti-neutrophil cytoplasmic antibody-associated vasculitis, *PR3-AAV* proteinase 3–anti-neutrophil cytoplasmic antibody-associated vasculitis, *Double-positive AAV* double-positive anti-neutrophil cytoplasmic antibody-associated vasculitis, *MPA* microscopic polyangiitis, *GPA* granulomatosis with polyangiitis, *EGPA* eosinophilic GPA, *eGFR* estimated glomerular filtration rate, *ESR* erythrocyte sedimentation rate, *CRP* C-reactive protein, *BVAS* birmingham vasculitis activity score, *ENT* ear nose and throat, *RRT* renal replacement therapy, *CYC* cyclophosphamide, *RTX* rituximab, *ESRD* end-stage renal disease

^a^*P* < 0.05 vs. MPO-AAV

^b^*P* < 0.05 vs. PR3-AAV

Idiopathic double-positive AAV patients had the lowest level of white blood cells among the three groups (6.88 ± 3.00) (*P* < 0.05). While the number of neutrophils and lymphocytes did not significantly differ from each group. The degree of anemia in idiopathic double-positive AAV (74.15 ± 40.10), reflected by the level of hemoglobin, was comparable to MPO-AAV (82.68 ± 20.04) and more severe than PR3-AAV (95.49 ± 23.47). The idiopathic double-positive AAV patients had the lowest level of platelets (183.71 ± 80.06) and the PR3-AAV patients had the highest level of platelets (330.39 ± 170.52) (*P* < 0.05). As for renal involvement, idiopathic double-positive AAV and MPO-AAV demonstrated more severe kidney damage than PR3-AAV patients, which was reflected by higher initial serum creatinine(Dp-AAV vs. MPO-AAV vs. PR3-AAV) (473.4 (167, 659) μmol/L vs. 329.5 (161.775, 562) μmol/L vs. 104 (64, 215.2) μmol/L) (*P* < 0.05) and lower initial eGFR (Dp-AAV vs. MPO-AAV vs. PR3-AAV) (7.42 (6.82, 30.78) mL/(min·1.73 m^2^) vs. 14 (7.3, 32.9025) mL/(min·1.73 m^2^) vs. 72.81 (35.495, 111.19) mL/(min·1.73 m^2^)) (*P* < 0.05). CRP levels for idiopathic double-positive AAV patients (8.94 (4.28, 67.75)) (median, IQR) was similar to MPO-AAV (22.30 (5.11, 76.75)) (median, IQR) (*P* > 0.05), which was much lower than the PR3-AAV group (74.80 (21.8, 128.50)) (median, IQR) (*P* < 0.05). Idiopathic double-positive AAV also had lower BVAS (14.71 ± 6.03) than PR3-AAV (19.73 ± 5.48) (*P* < 0.05), which was similar to MPO-AAV (17.45 ± 5.94) (*P* > 0.05). Furthermore, with regard to ENT involvement, similar to MPO-AAV patients (8.0%), idiopathic double-positive AAV (5.9%) had less ENT involvement than PR3-AAV (32.4%) (*P* < 0.05). Finally, the percentage of patients required for RRT at the commencement of treatment was 23.5% in the idiopathic double-positive AAV group.

### Renal histopathology

Seventy-four (25.9%) patients in the MPO-AAV group, 10 (27.0%) patients in the PR3-AAV group, and 7 (41.2%) patients in the idiopathic double-positive AAV group underwent renal biopsy. The pathological characteristics of the cohort are described in Table 2.

**Table 2:** The pathological characteristics

	MPO-AAV	PR3-AAV	double-positive AAV	*P* value
•No. of kidney biopsies, *n* (%)	74 (25.9%)	10 (27.0%)	7 (41.2%)	0.383
*•Berden classification*				
Focal, *n* (%)	11 (14.9%)	3 (30.0%)	1 (14.3%)	0.413
Crescentic, *n* (%)	22 (29.7%)	4 (40.0%)	3 (42.9%)	
Mixed, *n* (%)	20 (27.0%)	1 (10.0%)	3 (42.9%)	
Sclerotic, *n* (%)	21 (28.4%)	2 (20.0%)	0 (0%)	
*•Glomerular lesions*				
The proportion of normal glomeruli, % (median, IQR)	20 (0, 42)	43 (30, 85)	17 (14, 27)	0.021
The proportion of sclerotic glomeruli, % (median, IQR)	28 (13, 50)	6 (0, 48)	31 (0, 36)	0.170
The proportion of crescentic glomeruli, % (median, IQR)	42 (18, 60)	28 (0, 52)	44 (36, 50)	0.308
The proportion of cellular crescents, % (median, IQR)	0 (0, 11)	0 (0, 32)	5 (0, 50)	0.538
The proportion of cellular-fibrous crescents, % (median, IQR)	14 (0, 39)	0 (0, 32)	6 (0, 33)	0.346
Fibrinoid necrosis, *n* (%)	31 (41.9%)	1 (10.0%)	2 (28.6%)	0.141
Bowman’s capsule rupture, *n* (%)	22 (29.7%)	2 (20.0%)	4 (57.1%)	0.262
Thrombotic microangiopathy, *n* (%)	8 (10.8%)	0 (0%)	0 (0%)	0.788
•Interstitial infiltrates (*n*, 0/1/2/3)	0/32/34/8	0/7/3/0	0/2/3/2	0.263
•Tubulointerstitial lesions (*n*, 0/1/2/3)	0/26/34/14	0/7/3/0	0/3/2/2	0.205
*•Arteriolar lesions*				
Vascular fibrinoid necrosis, *n* (%)	3 (4.1%)	0 (0%)	1 (14.3%)	0.327
*•Immunofluorescence pattern, n (%)*				
C3 Number of negative	50 (67.6%)	6 (60.0%)	6 (85.7%)	0.683
Number of 1 +	10 (13.5%)	1 (10.0%)	1 (14.3%)	
Number of ≥ 2 +	14 (18.9%)	3 (30.0%)	0 (0%)	
C4 Number of negative	62 (83.8%)	10 (100.0%)	7 (100.0%)	1.000
Number of 1 +	6 (8.1%)	0 (0%)	0 (0%)	
Number of ≥ 2 +	6 (8.1%)	0 (0%)	0 (0%)	
C1q Number of negative	67 (90.5%)	6 (60.0%)	7 (100.0%)	0.076
Number of 1 +	3 (4.1%)	2 (20.0%)	0 (0%)	
Number of ≥ 2 +	4 (5.4%)	2 (20.0%)	0 (0%)	
IgA Number of negative	55 (74.3%)	7 (70.0%)	4 (57.1%)	0.365
Number of 1 +	14 (18.9%)	1 (10.0%)	2 (28.6%)	
Number of ≥ 2 +	5 (6.8%)	2 (20.0%)	1 (14.3%)	
IgG Number of negative	51 (68.9%)	7 (70.0%)	6 (85.7%)	0.909
Number of 1 +	10 (13.5%)	1 (10.0%)	1 (14.3%)	
Number of ≥ 2 +	13 (17.6%)	2 (20.0%)	0 (0%)	
IgM Number of negative	38 (51.4%)	3 (30.0%)	3 (42.9%)	0.599
Number of 1 +	11 (14.9%)	1 (10.0%)	1 (14.3%)	
Number of ≥ 2 +	25 (33.8%)	6 (60.0%)	3 (42.9%)	
*•Brix renal risk score, n (%)*				
Low-risk group	11 (14.9%)	7 (70.0%)^a^	0 (0.0%)^b^	0.002
Medium-risk group	30 (40.5%)	2 (20.0%)	4 (57.1%)	
High-risk group	33 (44.6%)	1 (10.0%)	3 (42.9%)	

^a^*P* < 0.05 vs. MPO-AAV

^b^*P* < 0.05 vs. PR3-AAV

In the idiopathic double-positive group, 1 out of 7 (14.3%) was classified as focal, 3 out of 7 (42.9%) were classified as crescentic, and 3 out of 7 (42.9%) were classified as mixed. There were no significant differences in the Berden classification among the three groups. The percentage of normal glomeruli tended to be lower in idiopathic double-positive AAV compared with the other two groups (*P* < 0.05), though the differences were not statistically significant when comparing every two groups in all three groups. The features of the glomerulus, including fibrinoid necrosis, Bowman’s capsule rupture, granulomatous lesions, and thrombotic microangiopathy, did not differ from each other in this cohort. Nevertheless, we noticed a relatively high level of Bowman’s capsule rupture of idiopathic double-positive AAV (57.1%). No significant differences in scores of interstitial infiltrates and tubulointerstitial lesions were found among the three groups. Neither were there significant differences in arteriolar fibrinoid necrosis and the intensity of deposits (C3, C4, C1q, IgA, IgG, and IgM) under an immunofluorescence microscope.

According to Brix’s Renal Risk Score (RRS), 33 out of 74 (44.6%) patients in the MPO-AAV group were classified into the high-risk group, while 7 out of 10 (70.0%) patients in the PR3-AAV group into the low-risk group. In the idiopathic double-positive group, 4 out of 7 (57.1%) patients were classified into the medium-risk group, and the percentage of the low-risk group was zero. The differences between the three groups were significant (*P* < 0.05). Both the proportions of the low-risk group in MPO-AAV patients and idiopathic double-positive AAV patients were lower than PR3-AAV (*P* < 0.05).

### Outcomes and survival analysis

Patient outcomes are shown in Table 1. The idiopathic double-positive AAV patients had the worst remission rate (58.8%) among the three groups (*P* < 0.05). The relapse rate of double-positive AAV (40.0%) was comparable with PR3-AAV (44.8%) (*P* > 0.05). Although there was a trend toward a higher relapse rate of idiopathic double-positive AAV (40.0%) compared with MPO-AAV (23.5%), this did not reach statistical significance (*P* > 0.05). The proportion of patients in idiopathic double-positive AAV who progressed to ESRD was 47.1% and that of MPO-AAV was 44.4%, where there was no statistical significance. The proportion of patients who progressed to ESRD in the PR3-AAV group (21.6%) was lower than that in the idiopathic double-positive AAV group (*P* < 0.05) without statistical significance. The idiopathic double-positive AAV group tended to have the lowest death rate among the three groups.

**Fig. 2:**
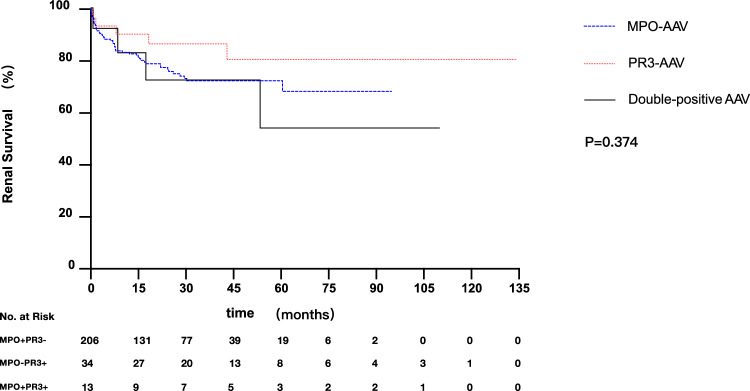
Kaplan–Meier Curves for the probability of renal survival according to ANCA specificity. Renal survival time (months) refers to the time since treatment. *P* = 0.192, MPO-AAV vs PR3-AAV; *P* = 0.738, MPO-AAV vs double-positive AAV; *P* = 0.307, PR3-AAV vs double-positive AAV

**Fig. 3:**
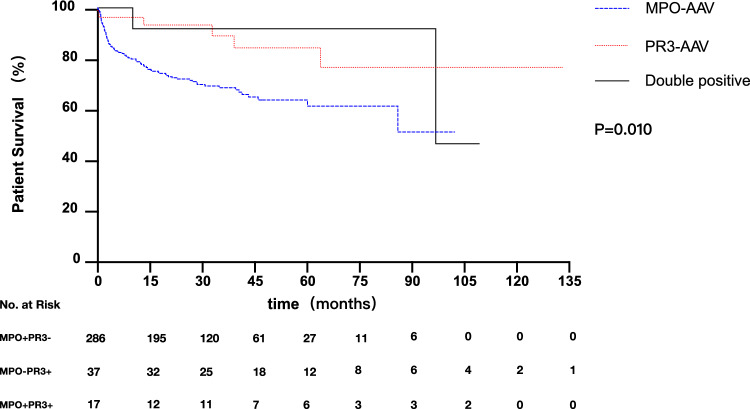
Kaplan–Meier Curves for the probability of patient survival according to ANCA specificity. Patient survival time (months) refers to the time since treatment. *P* = 0.014, MPO-AAV vs PR3-AAV; *P* = 0.062, MPO-AAV vs double-positive AAV; *P* = 0.993, PR3-AAV vs double-positive AAV

As shown in Fig. 2, idiopathic double-positive AAV tended to have worse long-term renal survival among the three groups, although there was no statistical difference in unadjusted analysis (*P* = 0.374). Long-term patient survival also varied among the three groups (*P* = 0.010) (Fig. 3). However, no significant difference in long-term patient survival was found when comparing idiopathic double-positive AAV with either MPO-AAV (*P* = 0.062) or PR3-AAV (*P* = 0.993).

## Discussion

To the best of our knowledge, our cohort included the largest number of double-positive AAV patients to compare outcomes of idiopathic double-positive AAV and single-positive AAV so far. The results of our study shed light on features of double-positive AAV. Demonstrating a hybrid disease phenotype, idiopathic double-positive AAV patients exhibited the propensity for renal deterioration of MPO-AAV and also exhibited the long-term risk of relapse tendency of PR3-AAV.

There was no evidence-based data about the incidence and prevalence of idiopathic double-positive AAV. Pearce, et al. mentioned 9 patients both PR3- and MPO-ANCA positive among a total of 1217 AAV patients [26]. Kim, et al. retrospectively summarized the characteristics of 8 biopsy-proven vasculitis patients with dual MPO- and PR3-ANCA positivity [27]. Chou et al. described the outcomes of 15 patients positive for both PR3- and MPO-ANCA. They found that 3 of 15 were finally diagnosed with AAV and presented with MPO-predominance [28]. Previous case reports about double-positive AAV were related to drug-induced AAV, membranous nephropathy, Henoch-Schonlein purpura, infective endocarditis, Moyamoya, mixed connective tissue disease and systemic lupus erythematosus [29]. We cautiously ruled out all possible secondary causes before our analysis.

As found in this study, idiopathic double-positive AAV exhibited composite characteristics of MPO-AAV and PR3-AAV. The main clinical phenotype of double-positive AAV was more like MPO-AAV. To elaborate on this, idiopathic double-positive AAV patients took features of MPO-AAV such as older age distribution, more severe anemia, lower BVAS, and CRP level, less ENT involvement, and more severe renal dysfunction, which was presented with higher initial serum creatinine and lower initial eGFR. Also, the prognosis of Brix’s RRS showed that the renal prognosis of PR3-AAV was better than that of MPO-AAV and idiopathic double-positive AAV. The similarity of age distribution and more severe renal dysfunction in idiopathic double-positive AAV is compatible with the study of Kim et al. [27]. Moreover, comparable with MPO-AAV, a majority of idiopathic double-positive AAV patients were diagnosed with MPA. There were 75% of double-positive AAV patients diagnosed with MPA in Kim's study [27]. We assumed that MPO-ANCA might play an important role in idiopathic double-positive AAV. In our cohort, there were more idiopathic double-positive patients being positive for p-ANCA and MPO-ANCA titers were higher compared with PR3-ANCA titers in these patients.

The relatively lower proportion of remission of idiopathic double-positive AAV cannot be ignored. Remission rates at 12 weeks and 1 year were also reported to be relatively lower in Kim's study [27]. Interestingly, idiopathic double-positive AAV patients have the lowest level of platelets in this study. Our group has demonstrated that treatment resistance was negatively associated with platelet levels in MPO-AAV previously [30]. Since idiopathic double-positive AAV showed a major MPO-AAV phenotype, we assume that the lower remission rate of idiopathic double-positive AAV had a possible correlation with its lower platelet counts.

Furthermore, it is noteworthy that idiopathic double-positive AAV patients had more probability of relapse, which was similar to PR3-AAV. The higher rate of relapse in PR3-AAV compared with MPO-AAV was verified in many other studies before [31–33]. Owing to the possibility of treatment resistance and risk of relapse, idiopathic double-positive AAV patients should be very carefully monitored.

Notably, idiopathic double-positive AAV had a worse renal prognosis. It was revealed that double-positive AAV had a relatively high rate of a requirement for RRT at presentation and a high proportion of patients progressed into ESRD. In addition, it appeared that idiopathic double-positive AAV had less normal glomeruli among the three groups. Since previous studies have suggested that the percentage of normal glomeruli is a reliable and independent predictor for renal outcome [19, 34], it is conceivable that idiopathic double-positive AAV patients have a high incidence of ESRD in our cohort. Also, the high ratio of Bowman’s capsule rupture in the idiopathic double-positive AAV group should not be ignored. Extra-capillary proliferation can occur via Bowman’s capsule rupture leading to infiltration of inflammatory cells and affecting the constitution of crescents [35]. Anqun Chen et al. observed that unimpaired Bowman's capsule could prevent CD8^+^ T cells from accessing podocytes. The podocyte can be destroyed by CD8^+^ T cells after Bowman's capsule rupture [36]. The higher initial serum creatinine, lower initial eGFR and relatively worse renal survival also suggest that idiopathic double-positive AAV patients might present with a “more deteriorative” renal function than PR3-AAV. No studies have analyzed the renal survival of idiopathic double-positive AAV before. Despite no significant differences in the death rate when comparing idiopathic double-positive AAV with MPO-AAV or PR3-AAV, there was no denying that the overall patient survival of idiopathic double-positive AAV was poor.

The mechanism for the production of dual-specificity ANCA remains elusive. NETS comprising extracellular DNA and histones, and neutrophil proteins such as myeloperoxidase, neutrophil elastase, or calgranulin can be released to the extracellular space after a particular process of neutrophil death called NETosis [37]. NETs not only have immunogenicity in vasculitis but also damage glomerular endothelial cells, podocytes, and parietal endothelial cells [38, 39]. Autoreactive T cells might recognize MPO and PR3 from NETs generated by neutrophils through antigen-presenting cells and then induce B cells to produce anti-MPO and anti-PR3 antibodies [40, 41]. Through animal experiments, we assumed that the production of MPO-ANCA and PR3-ANCA was related to DNA particles taken up by monocyte-derived dendritic cells (DCs) [42]. Moreover, MPO-ANCA and PR3-ANCA might not activate neutrophils in the same way [5]. In vitro,MPO-ANCA and PR3-ANCA cause neutrophils to be activated and go through an oxidative burst and degranulate [43]. PR3-ANCA can stimulate neutrophils more potently [44], and the sialylation level of PR3-ANCA correlated with the extent of the oxidative burst [45]. It needs further study to explore whether there exit synergistic effects of activating neutrophils between MPO-ANCA and PR3-ANCA in AAV or not. Further studies focusing on the disease pathogenesis of idiopathic double-positive AAV are needed. Double-positive AAV patients turned to be single-positive or negative for MPO-ANCA and PR3-ANCA during follow-up. It was reported that ANCA was considered to be a well-recognized diagnostic biomarker and the persistence of ANCA is related to the occurrence of relapse in the future [46, 47]. However, it was controversial to use ANCA titers to predict disease activities and/or guide therapy [3]. The usefulness of ANCA for follow-up was inconclusive. For these reasons, we highlighted the role of ANCA during the course of diagnosis rather than the ANCA persistence during follow-up.

There were certain limitations in our research. Firstly, the number of idiopathic double-positive AAV patients and the cases of patients who underwent kidney biopsy are relatively small, the firmness of the conclusion was circumscribed by which. Secondly, there was selection bias owing to our data coming from one single Chinese provincial center. Thus, further larger sample-sized research of idiopathic double-positive AAV conducted in multiple centers is required.

In summary, idiopathic double-positive AAV is a rare clinical entity with hybrid features of MPO-AAV and PR3-AAV. MPO-AAV is the “dominant” phenotype in idiopathic double-positive AAV. Except for the high risk of relapse like PR3-AAV, idiopathic double-positive AAV patients also experience unfavorable long-term renal prognoses like MPO-AAV.

## Data Availability

The original contributions presented in the study are included in the article. Further inquiries can be directed to the corresponding authors.
